# Association between serum-free thyroxine level and all-cause mortality in critically ill patients: a retrospective study from MIMIC-IV

**DOI:** 10.3389/fendo.2023.1164369

**Published:** 2023-05-25

**Authors:** Juan-Juan Dai, Ding-Fu Du, Gang Ma, Ming-Jie Jiang

**Affiliations:** ^1^ Department of Intensive Care Unit, State Key Laboratory of Oncology in South China, Sun Yat-Sen University Cancer Center, Collaborative Innovation Center for Cancer Medicine, Guangzhou, China; ^2^ Department of Head and Neck Surgery, State Key Laboratory of Oncology in South China, Sun Yat-sen University Cancer Center, Collaborative Innovation Center for Cancer Medicine, Guangzhou, China

**Keywords:** free thyroxine, mortality, tri-iodothyronine, ICU, MIMIC-IV

## Abstract

**Background:**

Low thyroxine (T4) levels have been observed in critically ill patients; however, controversial results regarding T4 supplemental therapy are reported. The association between serum free T4 (FT4) levels and mortality in critically ill patients has not been fully established and needs to be clarified.

**Methods:**

Data from the Medical Information Mart for Intensive Care (MIMIC)-IV were collected and analyzed. The association between FT4 level and 30-day mortality after ICU admission was analyzed using Kaplan–Meier curves, spline smoothing fitting, martingale residuals of the null Cox model, and restricted cubic spline (RCS). Logistic regression, Cox regression, and receiver operating characteristic curve (ROC) were used to uncover the relationship and predictive value of serum FT4 and 30-day mortality in critically ill patients.

**Results:**

In the final analysis, 888 patients were enrolled, and the serum FT4 levels were divided into four groups. A significant difference in 30-day mortality was observed between the four groups. Kaplan–Meier curves also presented significantly higher 30-day mortality in groups 1 and 2 (*p* < 0.0001). Further multivariance logistic regression showed that group 1 with FT4 levels lower than 0.7 μg/dl can predict 30-day mortality (odds ratio (OR) = 3.30, 95% confidence interval (CI) = 1.04–11.31). Spline smoothing fitting analysis showed a “V”-shaped line between 30-day mortality and FT4 level within 0–3 μg/dl. Further RCS analysis showed that the risk of death decreased rapidly as FT4 levels increased when serum FT4 levels were lower than 1.2 μg/dl and started to become flat afterward. The area under the ROC of the lower FT4 level to predict 30-day mortality was 0.833 (95% CI = 0.788–0.878). Both multivariant Cox regression and logistic regression showed that FT4 levels lower than 1.2 μg/dl can independently predict 30-day mortality when adjusted for other potential confounders (HR = 0.34, 95% CI = 0.14–0.82; OR = 0.21, 95% CI = 0.06–0.79, respectively), but its predictive power disappeared when adjusted for T3 or total T4.

**Conclusion:**

Serum FT4 levels were significantly negatively associated with 30-day mortality when they were lower than 1.2 μg/dl and could predict the risk of 30-day mortality. A higher FT4 level is potentially related to increased 30-day mortality.

## 1 Introduction

Thyroid hormone is critical in regulating physiological processes, including growth and metabolism. After being produced in the thyroid, thyroxine (T4) was then released to the peripheral blood and converted to tri-iodothyronine (T3) in various tissues to exert its function. Thyroid function can easily be affected by a wide variety of factors, such as malnutrition, sepsis, major injuries, etc. As a result, dysfunction of the thyroid is commonly seen in critically ill patients (1). In particular, low T3 levels combined with thyroid-stimulating hormone (TSH) levels in the reference range have been referred to as low T3 syndrome or nonthyroidal illness syndrome (NTIS), which has been recognized as a predictor for poor prognosis in many diseases, such as sepsis (2), chronic lymphocytic leukemia (3), chronic hemodialysis (4), COVID-19 (5), and stress cardiomyopathy (6).

Low T4 levels in patients with NTIS are often observed in more critically ill patients, and they were originally thought to be associated with worse outcomes in patients (7). Low T4 can also suggest the lower function of the thyroid such as hypothyroidism, which in its severe case is also a lethal condition in the ICU (1). Notably, although hormone supplemental therapy is recommended in patients with primary hypothyroidism, there are controversial results in terms of T4 supplements in NTIS (8–10). Thus, it is important to decipher the relationship between serum free T4 (FT4) levels and mortality in critically ill patients, which is the goal of this study.

## 2 Materials and methods

### 2.1 Data source and study population

Data used in this study were extracted from Medical Information Mart for Intensive Care (MIMIC)-IV v. 2.1, a large publicly available database that includes information on over 50,000 ICU admissions at the Beth Israel Deaconess Medical Center (Boston, Massachusetts) from 2008 to 2019. Author Dai has passed the online exam to get access to the database (Certification number: 53091918).

Patients with serum FT4 levels in the first 24 h of ICU admission were selected as the study population. The exclusion criteria were as follows: (1) patients with an age of under 18 years; (2) not the first ICU stay record; and (3) thyroxine usage, including levothyroxine sodium, thyroid, liothyronine, and liothyronine sodium, before ICU admission. We then divided patients into four groups according to the reference range of serum FT4 levels: patients with FT4 levels below/within/over the reference range; patients with normal FT4 levels were further divided into two groups at the cut point of 1.2 μg/dl, which is the median value of reference range.

### 2.2 Data extraction

PostgreSQL tools (v.14, PostgreSQL Global Development Group, Berkley, California, USA) and Navicat Premium 15 were used for the data extraction. Lab parameters, vital signs, disease severity scores within 24 h of admission to the ICU, and thyroxine usage before ICU admission were extracted. The diagnosis information was extracted by the International Classification of Disease (ICD) code ICD-9. All information was then matched according to ICU stay identity (ID). The variables extracted include the following: (1) general information, including age, gender, weight, and height; (2) lab parameters within the first 24 h of ICU admission, including FT4, T4, T3, calculated T4 index, TSH, TPOAb, parathyroid hormone, glucose, hemoglobin, red blood cell (RBC), white blood cell (WBC), and platelets; (3) vital signs within the first 24 h of ICU admission, including temperature, heart rate, SpO_2_, mean blood pressure (MBP), systolic blood pressure (SBP), and diastolic blood pressure (DBP); (4) disease severity scores within the first 24 h of ICU admission, including sequential organ failure assessment (SOFA), oxford acute severity of illness score (OASIS), acute physiology score (APS)III, and simplified acute physiology score (SAPS)II; and (5) incidence of comorbidities, including heart failure, cardiogenic shock, respiratory failure, kidney disease, diabetes, thyroid disease, adrenal gland disease, parathyroid disease, hypertension, and hypotension.

### 2.3 Statistical analysis

Statistical analysis was performed by R software 4.2.1. In the baseline characteristics, non-normally distributed continuous variables were presented as median with an interquartile range and tested using the Kruskal–Wallis test. While the normally distributed ones were presented as mean ± standard deviation, and *t*-tests were used for comparison between groups. Categorical variables were presented as counts and percentages, which were compared by Pearson’s Chi-squared test. Cox proportional hazards regression analyses and logistic regression analyses were used to assess the relationship between serum FT4 level and 30-day mortality. Receiver operating characteristic curves (ROCs) were used to evaluate the prediction effect for serum FT4 levels in critically ill patients. The Kaplan–Meier curves (log-rank test) were used to compare the 30-day survival rate between patients with different FT4 levels. *p <*0.05 was considered statistically significant. Spline smoothing fitting and martingale residuals of the null Cox model was used to explore the crude relationship between FT4 level and 30-day mortality. Restricted cubic spline (RCS) with five knots was used to flexibly model the association of FT4 level with 30-day mortality.

## 3 Results

### 3.1 Basic characteristics of patients

For the final analysis, 888 patients with serum FT4 levels in the first 24 h of ICU admission were selected from the MIMIC-IV database and categorized into four groups according to serum FT4 level reference range (0.7–1.8 μg/dl) and the median value of reference range (1.2 μg/dl), with 58 patients in group 1 (FT4 < 0.7 μg/dl), 488 patients in group 2 (0.7 ≤ FT4 ≤ 1.2 μg/dl), 282 patients in group 3 (1.2 < FT4 ≤ 1.8 μg/dl), and 60 patients in group 4 (FT4 > 1.8 μg/dl). A univariate analysis of the basic characteristics of patients by serum FT4 level is shown in **Table 1**. The mean age of patients is 66.12 ± 17.14 years, while patients in group 4 with the highest FT4 level have the lowest mean age of 59.62 ± 17.75 years (*p* < 0.05). As expected, serum T3, T4, and calculated T4 index increased with FT4, while TSH declined with it (*p* < 0.05). Notably, both 7- and 30-day mortality decreased as the FT4 level increased, with a slight increase in group 4 (*p* < 0.05). Similarly, patients with higher FT4 levels had lower severity scores, including APSIII, SAPSII, OASIS, and SOFA scores, except for patients in group 4 (*p* < 0.05). Heart failure, respiratory failure, and kidney diseases, including kidney failure, are the most frequent comorbidities in the entire patient collective. Although incidences of comorbidities of heart failure, cardiogenic shock, respiratory failure, kidney disease, thyroid disease, and hypertension were significantly different between the four groups (*p* < 0.05), no obvious association between them and 7- or 30-day mortality was observed.

**Table 1:** Basic characteristics of patients according to FT4 levels.

Variables	FT4 level (μg/dl)				*p*-value
	<0.7 (*n* = 58)	≧0.7; ≦1.2 (*n* = 488)	>1.2; ≤1.8 (*n* = 282)	>1.8 (*n* = 60)	
Age (years; median (Q1–Q3))	68.78 (57.43, 78.67)	68.71 (56.29, 78.83)	67.08 (54.70, 80.46)	61.35 (45.24, 73.52)	0.0399
Sex (*n* (%))					
Female	36 (62.07)	264 (54.10)	150 (53.19)	33 (55.00)	0.6667
Male	22 (37.93)	224 (45.90)	132 (46.81)	27 (45.00)
Height (cm)	168 (157, 175)	168 (160, 175)	168 (160, 175)	168 (160, 173)	0.94
Weight (kg)	77.2 (61.0, 100.9)	74.5 (62.2, 90.6)	74.7 (62.0, 92.5)	70.7 (58.7, 84.3)	0.3639
Comorbidity (*n* (%))					
Heart failure	16 (27.59)	173 (35.45)	116 (41.13)	32 (53.33)	0.0108
Cardiogenic shock	1 (1.72)	27 (5.53)	32 (11.35)	6 (10.00)	0.0068
Respiratory failure	32 (55.17)	179 (36.68)	78 (27.66)	20 (33.33)	0.0005
Kidney disease	39 (67.24)	283 (57.99)	125 (44.33)	28 (46.67)	0.0003
Diabetes	18 (31.03)	179 (36.68)	92 (32.62)	17 (28.33)	0.426
Thyroid disease	48 (82.76)	193 (39.55)	138 (48.94)	47 (78.33)	<0.0001
Adrenal gland disease	3 (5.17)	23 (4.71)	20 (7.09)	1 (1.67)	0.2928
Parathyroid disease	2 (3.45)	13 (2.66)	4 (1.42)	2 (3.33)	0.6102
Hypertension	25 (43.10)	196 (40.16)	114 (40.43)	13 (21.67)	0.0371
Hypotension	4 (6.90)	68 (13.93)	42 (14.89)	7 (11.67)	0.415
Vital signs					
Temperature (°C)	36.67 (36.33, 37.10)	36.67 (36.36, 37.00)	36.67 (36.39, 37.00)	36.72 (36.47, 36.94)	0.8678
Heart rate (bpm)	84 (71, 104)	87 (72, 104)	90 (75, 108)	99 (77, 119)	0.0047
SpO_2_ (%)	97 (95, 100)	98 (95, 100)	98 (96, 100)	97 (95, 99)	0.2148
DBP (mmHg)	68 (56, 80)	68 (56, 79)	71 (61, 86)	72 (60, 87)	0.0028
MBP (mmHg)	84 (70, 100)	81 (69, 93)	86 (74, 101)	88 (75, 99)	0.0017
SBP (mmHg)	120 (105, 143)	119 (103, 138)	126 (110, 145)	124 (108, 142)	0.0074
Laboratory parameters					
T3 (μg/dl)	45 (34, 52)	62 (48, 88)	77 (60, 106)	151 (116, 234)	<0.0001
T4 (μg/dl)	2.15 (1.8, 2.8)	5.15 (4.28, 6.23)	7.75 (6.75, 8.95)	10.65 (8.55, 13.78)	<0.0001
Calculated T4 index (μg/dl)	1.9 (1.2, 2.8)	5.4 (5.23, 5.65)	9.25 (8.45, 9.83)	15.9 (13.5, 22.3)	<0.0001
TSH (μIU/ml)	9.6 (3.13, 28.75)	2.95 (1.1, 6.1)	1.9 (0.85, 4.48)	1.65 (0.21, 5.00)	<0.0001
TPOAb (IU/ml)	213 (192, 234)	11 (11, 11)	17 (17, 17)	877 (450.5, 933)	0.2576
Glucose (mg/dl)	125 (98.25, 160)	132 (105, 172)	125 (100, 164)	133 (94.75, 163.5)	0.4449
Hemoglobin (g/dl)	10.0 (8.3, 11.5)	10.7 (9.0, 12.6)	11.4 (9.8, 12.9)	11.75 (10.375, 13.4)	<0.0001
Platelets (×10^9^/L)	197.5 (146.25, 301.25)	195 (136.5, 266)	227.5 (162.25, 290.5)	210.5 (167.75, 269.5)	0.001
RBC (×10^12^/L)	3.38 (0.91)	3.63 (0.85)	3.86 (0.80)	3.99 (0.83)	<0.0001
WBC (×10^9^/L)	9.75 (7.925, 13.35)	9.75 (6.8, 14.325)	9.4 (6.9, 14.1)	9.85 (6.875, 13.575)	0.9361
Severity score					
SOFA	7 (4, 10)	6 (3, 9)	4 (2, 7)	4.5 (2, 7)	<0.0001
APS III	63.5 (46.75, 87.5)	55 (40, 75)	49 (36, 64.75)	51.5 (38, 73)	0.0002
OASIS	37 (29.25, 41.75)	33 (26, 41)	32 (26, 37)	32 (25.75, 38.25)	0.0193
SAPS II	40.5 (30.25, 53.75)	38 (30, 49)	35 (26.25, 44)	33.5 (27.75, 43.25)	0.0012
Outcomes					
7-day mortality (*n* (%))	11 (18.97)	78 (15.98)	22 (7.80)	6 (10.00)	0.0053
30-day mortality (*n* (%))	20 (34.48)	108 (22.13)	35 (12.41)	8 (13.33)	0.0001

### 3.2 Association between serum FT4 level on ICU admission and 30-day mortality in a critically ill patient

**Figure 1:**
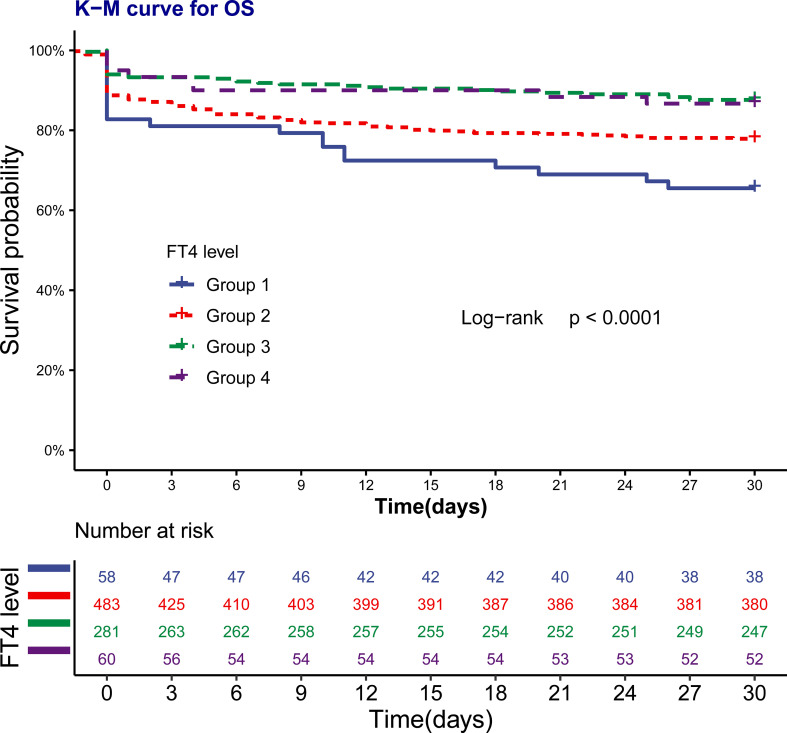
Kaplan-Meier curve for 30-day survival by FT4 levels. The number at risk represents the patients’ number in each FT4 levels at the start of each time period.

We found that 30-day mortality was the highest in patients with the lowest FT4 level and showed a declined trend as FT4 increased (*p* < 0.05). Kaplan–Meier curves also presented a significantly higher risk of death over 30 days in groups 1 and 2 (*p* < 0.0001; **Figure 1**).

To further explore the association between serum FT4 level and mortality, we first applied the logistic regression analysis (**Table 2**). Univariate logistic regression analysis showed that group 1 had a high predictive power for 30-day mortality (odds ratio (OR) = 3.42, 95% confidence interval (CI) = 1.40–9.02), while other groups did not. This predictive power also existed in multivariate logistic regression (OR = 3.30, 95% CI = 1.04–11.31), after adjusting for potential variables related to death that were statistically significant in univariable logistic regression analysis, including age, heart rate, temperature, SpO_2_, DBP, MBP, SBP, hemoglobin, platelets, RBC, WBC, SOFA, APSIII, OASIS, SAPSII, the incidence of cardiogenic shock, respiratory failure, and kidney disease (listed in **Supplementary Table S1**, *p* < 0.05). Notably, T3 and T4 dropped out of multivariate logistic regression due to a limited number (*n* = 35 for patients with both T3 and T4 results). When adjusting with serum T3 (*n* = 162) or T4 (*n* = 151) levels, the predictive power of FT4 disappeared, as listed in **Table 2**. These results indicated that although the FT4 level can predict 30-day mortality, its predictive power is largely affected by serum T3 and T4 levels.

**Table 2:** Logistic regression analyses for 30-day mortality of critically ill patients according to serum FT4 level on ICU admission.

Groups	Nonadjusted		Adjust 1		Adjust 2		Adjust 3	
	OR (95% CI)	*p*-value	OR (95%CI)	*p*-value	OR (95%CI)	*p*-value	OR (95%CI)	*p*-value
Group 1	3.42 (1.40–9.02)	0.0088	3.30 (1.04–11.31)	0.0481	0.68 (0.08–6.78)	0.7223	1.15 (0.06–26.65)	0.9272
Group 2	1.85 (0.90–4.31)	0.1203	2.09 (0.81–6.10)	0.1482	0.41 (0.07–3.31)	0.3484	0.81 (0.10–9.10)	0.8469
Group 3	0.92 (0.42–2.24)	0.8450	1.65 (0.61–5.01)	0.3487	0.48 (0.07–3.96)	0.4445	0.19 (0.01–2.31)	0.2004
Group 4	1.0 (ref)	–	1.0 (ref)	–	1.0 (ref)	–	1.0 (ref)	–

Group 1: Admission serum FT4 < 0.7 μg/dl; group 2: admission serum FT4 ≥ 0.7; ≤ 1.2 μg/dl; group 3: admission serum FT4 > 1.2; ≤ 1.8 μg/dl; group 4: admission serum FT4 > 1.8 μg/dl.

Adjust 1 for age, heart rate, temperature, SpO_2_, DBP, MBP, SBP, hemoglobin, platelets, RBC, WBC, SOFA, APS III, OASIS, SAPS II, cardiogenic shock, respiratory failure, and kidney disease. *n* = 888.

Adjust 2 for serum T3 level. *n* = 162.

Adjust 3 for serum T4 level. *n* = 151.

**Figure 2:**
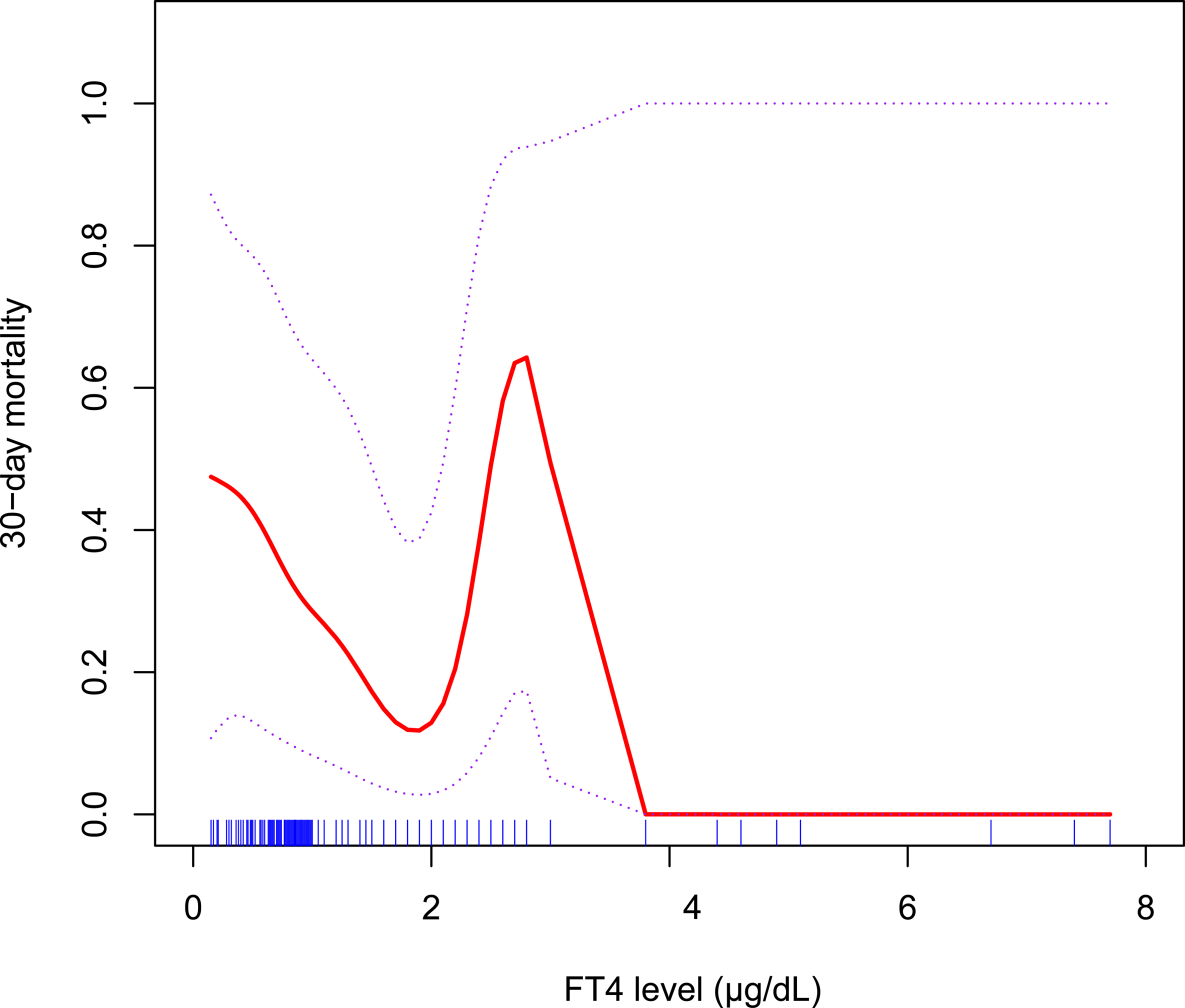
A spline smoothing fitting for the relationship between serum FT4 level and the risk of 30-day mortality before any adjustment.

Since a slight increase in 30-day mortality in group 4 was observed, we explored the crude relationship between FT4 and 30-day mortality by using the spline smoothing fitting before any adjustment (**Figure 2**). Interestingly, the result showed that when the FT4 level was less than 1.9 μg/dl, 30-day mortality was negatively associated with FT4 and positively associated until the FT4 level reached 3 μg/dl, suggesting that the relationship between FT4 level and 30-day mortality may not be strictly linear. In addition, the mortality of patients decreased rapidly when FT4 is more than 3 µg/dl. However, the number of patients with FT4 level of more than 3 µg/dl was rather small; this trend may not be statistically or clinically significant and need further verification. Martingale residuals of the null Cox model also showed a potential nonlinear relationship between FT4 level and 30-day mortality (**Supplementary Figure S1**).

**Figure 3:**
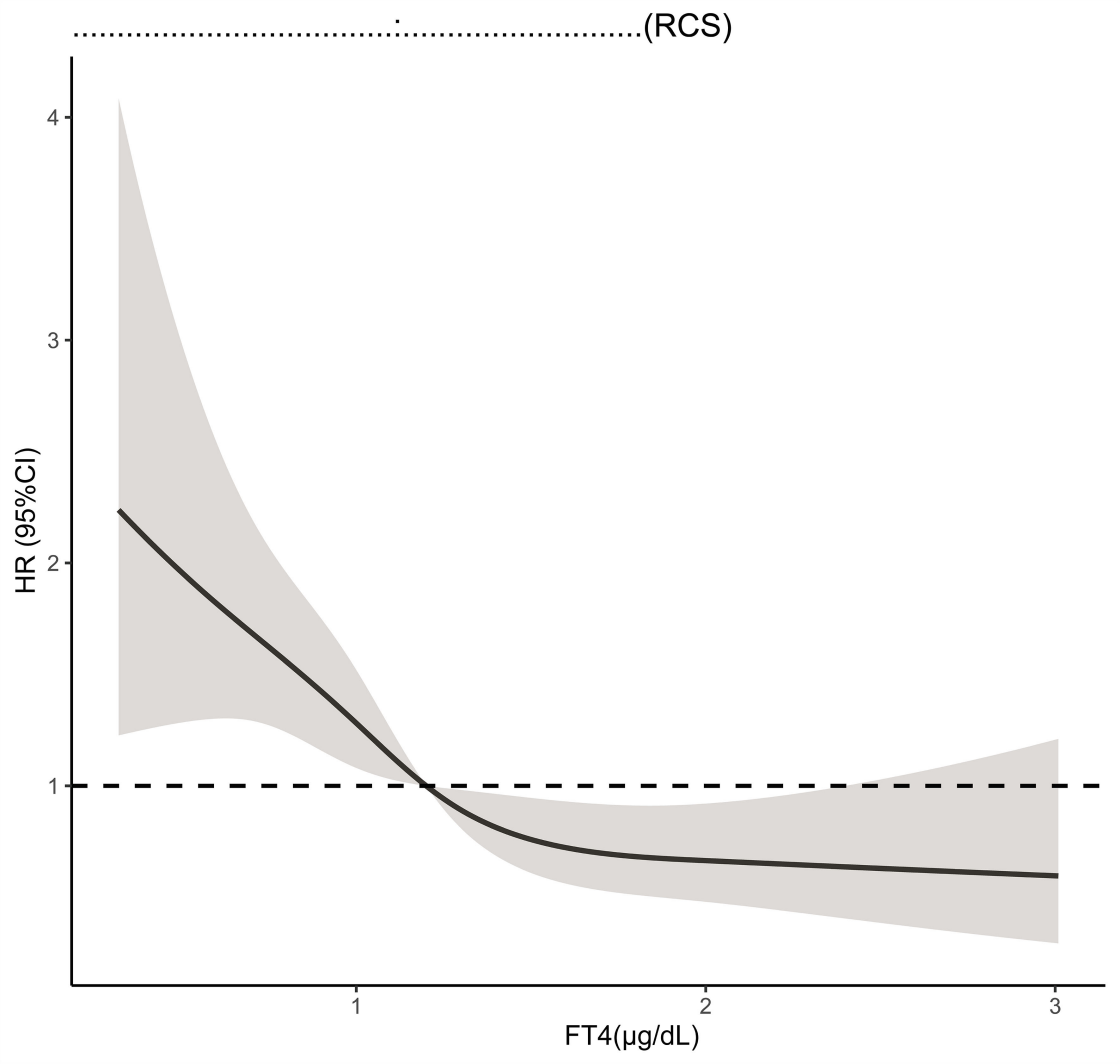
Restricted cubic spline model for the association of serum FT4 level with all cause 30-day mortality. Hazard ratios are indicated by solid lines and 95% CIs by shaded areas.

Next, the restricted cubic spline (RCS) was applied to model the potential nonlinear relationship between FT4 level and 30-day mortality with 5 knots (**Figure 3**). The risk of 30-day mortality declined rapidly as the FT4 level increased when the FT4 level was less than 1.2 μg/dl and became relatively flat when the FT4 level was greater than 1.2 μg/dl (*p* for nonlinearity = 0.0762), indicating that the FT4 level can better predict 30-mortality when it is less than 1.2 μg/dl.

### 3.3 The predictive power of lower FT4 level on 30-day mortality

**Figure 4:**
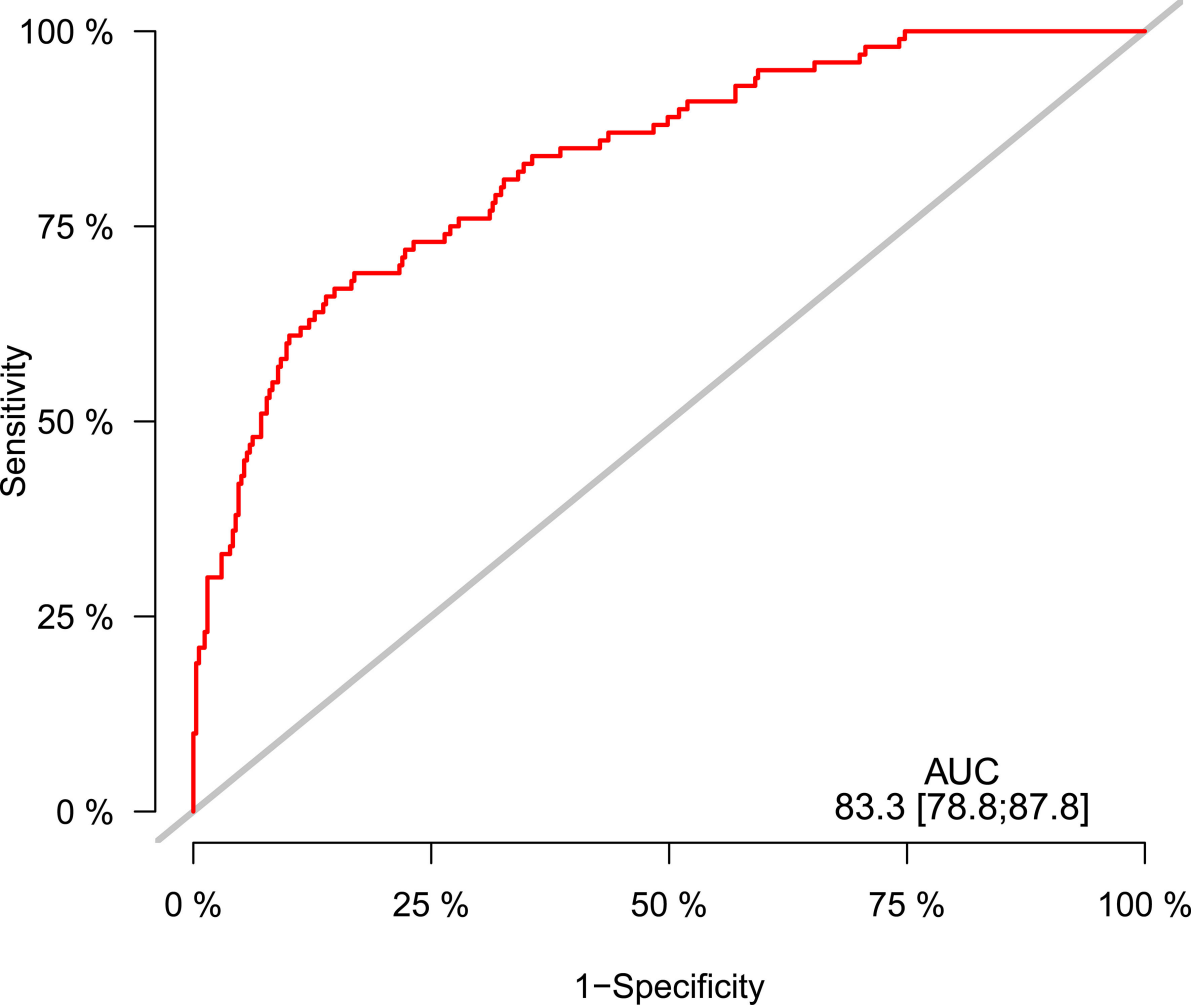
Receiver operating characteristic curve for predicting 30-day mortality in critically ill patients.

Considering the result of the RCS model, we next sought to explore the predictive power of FT4 levels under 1.2 μg/dl on 30-day mortality. Both Cox regression analysis and logistic regression analysis were performed in patients with FT4 levels less than 1.2 μg/dl (**Tables 3**, **4**). In the univariant analysis of both models, the FT4 level showed a significant predictive power on 30-day mortality (HR = 0.3228, 95% CI = 0.1413–0.7372; OR = 0.2403, 95% CI = 0.0864–0.6734, respectively). Thus, when adjusting for potential confounders listed, multivariable models did not appreciably change the results that the univariate risk estimated (HR = 0.3382, 95% CI = 0.1393–0.8210; OR = 0.2147, 95% CI = 0.0577–0.7882, respectively). Nevertheless, similar to the prior analysis, after adjusting for T3 or T4, the predictive power of FT4 disappeared. In addition, older age, a faster heart rate, a lower platelet count, and a higher APSIII score are independent risk factors for higher 30-day mortality in the multivariant analysis of both Cox regression and logistic regression (**Tables 3**, **4**). Of note, the area under the receiver operating characteristic curve (AUROC) of the FT4 level to predict 30-day mortality was 0.833 (95% CI = 78.8–87.8; **Figure 4**).

**Table 3:** Cox regression analysis for 30-day mortality in patients with lower FT4 levels.

Parameters	Nonadjusted		Adjust 1		Adjust 2		Adjust 3	
	HR (95% CI)	*p*-value	HR (95% CI)	*p*-value	HR (95% CI)	*p*-value	HR (95% CI)	*p*-value
Age	1.03 (1.01–1.04)	0.0002	1.03 (1.01–1.05)	0.0010				
T3	0.97 (0.94–0.99)	0.0111			1.03 (0.94–1.00)	0.0254		
T4	0.67 (0.47–0.95)	0.0254					1.78 (0.25–1.27)	0.1650
FT4	0.32 (0.14–0.74)	0.0073	0.34 (0.14–0.82)	0.0166	1.02 (0.15–6.27)	0.9865	0.3 (0.06–112.49)	0.6290
Heart rate	1.01 (1.00–1.02)	0.0113	1.01 (1.00–1.02)	0.0042				
MBP	0.99 (0.98–1.00)	0.0347						
SBP	0.99 (0.98–0.99)	0.0005						
Hemoglobin	0.83 (0.76–0.90)	<0.0001						
Platelets	0.99 (0.99–1.00)	<0.0001	1.00 (0.99–1.00)	0.0046				
RBC	0.63 (0.50–0.80)	0.0001						
SOFA	1.22 (1.17–1.28)	<0.0001						
APSIII	1.04 (1.03–1.04)	<0.0001	1.02 (1.01–1.04)	0.0010				
OASIS	1.08 (1.06–1.10)	<0.0001						
SAPSII	1.06 (1.04–1.07)	<0.0001						
Cardiogenic shock	2.98 (1.59–5.57)	0.0007						
Respiratory failure	2.91 (1.94–4.37)	<0.0001	1.64 (1.03–2.61)	0.0356				
Kidney disease	3.22 (1.95–5.31)	<0.0001						

Adjust 1 for age, heart rate, MBP, SBP, hemoglobin, platelets, RBC, SOFA, APS III, OASIS, SAPS II, the incidence of cardiogenic shock, respiratory failure, and kidney disease. *n* = 437.

Adjust 2 for serum T3 level. *n* = 77.

Adjust 3 for serum T4 level. *n* = 73.

**Table 4:** Logistic regression analyses for 30-day mortality in patients with lower FT4 levels.

Parameters	Nonadjusted		Adjust 1		Adjust 2		Adjust 3	
	OR (95% CI)	*p*-value	OR (95% CI)	*p*-value	OR (95% CI)	*p*-value	OR (95% CI)	*p*-value
Age	1.03 (1.02–1.05)	0.0001	1.05 (1.02–1.07)	0.0001				
T3	0.96 (0.93–0.99)	0.0108			0.96 (0.92–0.99)	0.0269		
T4	0.63 (0.40–0.92)	0.0249					0.54 (0.20–1.21)	0.1697
FT4	0.24 (0.09–0.67)	0.0063	0.21 (0.06–0.79)	0.0205	0.78 (0.08–8.46)	0.8339	2.34 (0.03–181.19)	0.6964
Heart rate	1.01 (1.00–1.02)	0.0177	1.01 (1.00–1.03)	0.0298				
MBP	0.99 (0.98–1.00)	0.0413						
SBP	0.98 (0.97–0.99)	0.0008						
Hemoglobin	0.81 (0.73–0.89)	<0.0001						
Platelets	0.99 (0.99–1.00)	<0.0001	1.00 (0.99–1.00)	0.0065				
RBC	0.59 (0.44–0.78)	0.0002						
SOFA	1.27 (1.20–1.36)	<0.0001						
APSIII	1.04 (1.03–1.05)	<0.0001	1.03 (1.01–1.05)	0.0067				
OASIS	1.10 (1.07–1.13)	<0.0001						
SAPSII	1.07 (1.05–1.09)	<0.0001						
Cardiogenicshock	4.50 (1.81–11.50)	0.0012	3.44 (1.15–10.49)	0.0269				
Respiratory failure	3.33 (2.10–5.34)	<0.0001						
Kidney disease	3.67 (2.18–6.48)	<0.0001						

Adjust 1 for age, heart rate, temperature, SpO_2_, DBP, MBP, SBP, hemoglobin, platelets, RBC, WBC, SOFA, APS III, OASIS, SAPS II, the incidence of cardiogenic shock, respiratory failure, and kidney disease. *n* = 437.

Adjust 2 for serum T3 level. *n* = 77.

Adjust 3 for serum T4 level. *n* = 73.

## 4 Discussion

In our study, the association between serum FT4 level on ICU admission and 30-day mortality was retrospectively analyzed using data collected from the MIMIC-IV database. A negative association between FT4 and 30-day mortality was observed when the FT4 level is less than 1.2 μg/dl, and although FT4 can be a prognostic factor for 30-day mortality, its prognostic power is largely affected by serum T3 and T4 levels. Importantly, a “V”-shaped curve was observed between 30-day mortality and FT4 level within 0–3 μg/dl, indicating the potentially harmful effect of overdosed T4 supplemental therapy.

Low FT4 levels can occur in patients with NTIS and primary hypothyroidism, which can be hard to discriminate in the context of critically ill conditions. Nonetheless, low FT4 levels in NTIS could reflect the occurrence of severe multiorgan malfunction and are associated with a worse outcome. Previous studies mainly focused on the T3 level and did not establish a negative relation between serum FT4 level and mortality (11–16). In a retrospective study, FT4 levels were found associated with 28-day mortality in patients with sepsis; however, no further analysis regarding the prognostic value of FT4 was performed (17). Despite all factors related, our result showed a clear prognostic power of the FT4 level on 30-day mortality. The risk of death decreased as the serum FT4 level increased when the FT4 level was less than 1.2 μg/dl, which may indicate the need to use a T4 supplement in patients with significantly low FT4.

Although hormone supplemental therapy is recommended in patients with primary hypothyroidism, there are controversial results in terms of T4 supplements in NTIS (8–10). Although a beneficial effect on cardiac performance has been observed in patients with idiopathic dilated cardiomyopathy (10), levothyroxine usage in ICU patients with low T4 concentrations showed no obvious benefits (8), and even worse, a slightly increased mortality has been observed in patients with acute renal failure treated with levothyroxine (9). Notably, we did observe an increase in 30-day mortality when the FT4 level is within the range of 1.9–3 μg/dl in the spline smoothing fitting analysis. However, when analyzing patients with FT4 levels between 1.2 and 3 μg/dl (*n* = 342) or 1.9 and 3 μg/dl (*n* = 46), no significant predictive power of FT4 was observed either in univariant and multivariant logistic analyses (data not shown), probably due to the limited number of patients enrolled. Notably, previous studies used certain dosages of T4 supplements and mostly aimed to maintain FT4 levels in the normal reference. However, it is possible that a higher FT4 level could increase 30-day mortality, although it is under the normal reference range of FT4. A more sophisticated study should be designed to clarify at what level thyroid hormones should be maintained to achieve better outcomes.

## Data availability statement

The raw data supporting the conclusions of this article will be made available by the authors, without undue reservation.

## Ethics statement

Ethical review and approval were not required for the study on human participants in accordance with local legislation and institutional requirements. Written informed consent for participation was not required for this study in accordance with national legislation and institutional requirements.

## Author contributions

J-JD conceived and performed the analysis. J-JD and D-FD are responsible for data interpretation and manuscript writing. GM and M-JJ supervised data processing and revised the manuscript. All authors contributed to the article and approved the submitted version.
