# Supplementary material for: Association between serum-free thyroxine level and all-cause mortality in critically ill patients: a retrospective study from MIMIC-IV
Source: Front Endocrinol (Lausanne). 2023 May 25;14:1164369. doi: 10.3389/fendo.2023.1164369 (PMC10248474; doi:10.3389/fendo.2023.1164369)
Supplement: Supplementary file 1 [file DataSheet_1.docx]

Supplementary Material

Association between serum free thyroxine level and all-cause mortality in critically ill patients: a retrospective study from MIMIC-IV

**Juan-juan Dai*, Ding-fu Du*, Gang Ma, Ming-jie Jiang**

**Correspondence:** Ming-jie Jiang and Gang Ma

Jiangmj1@sysucc.org.cn (MJ Jiang) and magang@sysucc.org.cn (G Ma)

# Supplementary Data

Supplementary Material contains 1 supplementary table and 1 supplementary figure.

## Supplementary Figure


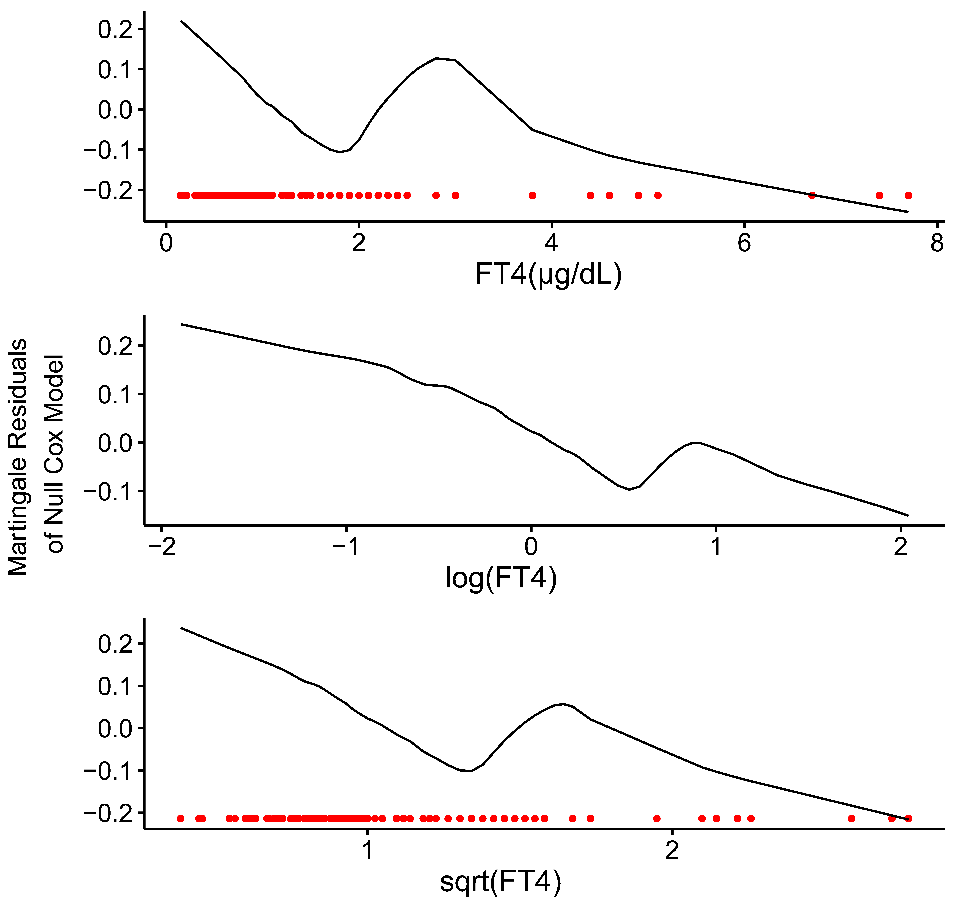


**Supplementary Figure 1.** Martingale residuals of null cox model showing the relationship between serum FT4 level and 30-day mortality.

## Supplementary table

Supplementary table 1. Univariate logistic regression analysis of critically ill patients.

| Parameters | Univariate analysis | | |
| --- | --- | --- | --- |
|  | OR | 95%CI | P value |
| Age | 1.0262 | 1.0153-1.0377 | <0.0001 |
| T3 | 0.9720 | 0.9522-0.9883 | 0.0028 |
| T4 | 0.7445 | 0.5944-0.9032 | 0.0056 |
| FT4 | 0.4733 | 0.2998-0.7180 | 0.0009 |
| Temperature | 0.7716 | 0.6638-0.8963 | 0.0007 |
| Heart rate | 1.0085 | 1.0018-1.0151 | 0.0120 |
| SpO2 | 0.9440 | 0.9099-0.9787 | 0.0017 |
| DBP | 0.9891 | 0.9799-0.9981 | 0.0203 |
| MBP | 0.9883 | 0.9793-0.9970 | 0.0099 |
| SBP | 0.9893 | 0.9825-0.9959 | 0.0017 |
| Hemoglobin | 0.8132 | 0.7543-0.8746 | <0.0001 |
| Platelets | 0.9948 | 0.9929-0.9966 | <0.0001 |
| RBC | 0.6300 | 0.5111-0.7727 | <0.0001 |
| WBC | 1.0227 | 1.0040-1.0429 | 0.0190 |
| SOFA | 1.2769 | 1.2215-1.3377 | <0.0001 |
| OASIS | 1.1136 | 1.0915-1.13733 | <0.0001 |
| SAPSII | 1.0671 | 1.0538-1.0812 | <0.0001 |
| Cardiogenic shock | 1.9320 | 1.0896-3.3175 | 0.0198 |
| Respiratory failure | 3.1225 | 2.2206-4.4080 | <0.0001 |
| Kidney disease | 3.8377 | 2.6252-5.7282 | <0.0001 |
|  |  |  |  |
